# Variations in maternal vitamin A intake modifies phenotypes in a mouse model of 22q11.2 deletion syndrome

**DOI:** 10.1002/bdr2.1709

**Published:** 2020-05-20

**Authors:** Gelila Yitsege, Bethany A. Stokes, Julia A. Sabatino, Kelsey F. Sugrue, Gabor Banyai, Elizabeth M. Paronett, Beverly A. Karpinski, Thomas M. Maynard, Anthony‐S. LaMantia, Irene E. Zohn

**Affiliations:** ^1^ Department of Anatomy and Cell Biology The George Washington University School of Medicine and Health Sciences Washington District of Columbia USA; ^2^ Institute for Neuroscience The George Washington University School of Medicine and Health Sciences Washington District of Columbia USA; ^3^ Center for Neuroscience Research Children’s Research Institute, Children’s National Medical Center Washington District of Columbia USA; ^4^ Center for Genetic Medicine Children’s Research Institute, Children’s National Medical Center Washington District of Columbia USA; ^5^ Fralin Biomedical Research Institute at Virginia Tech Carilion School of Medicine Roanoke Virginia USA; ^6^ Department of Biological Sciences Virginia Tech Blacksburg Virginia USA

**Keywords:** 22q11.2 deletion syndrome, Retinoid acid, vitamin A, Dysphagia, Gene‐environment interaction

## Abstract

**Background:**

Vitamin A regulates patterning of the pharyngeal arches, cranial nerves, and hindbrain that are essential for feeding and swallowing. In the *LgDel* mouse model of 22q11.2 deletion syndrome (22q11DS), morphogenesis of multiple structures involved in feeding and swallowing are dysmorphic. We asked whether changes in maternal dietary Vitamin A intake can modify cranial nerve, hindbrain and pharyngeal arch artery development in the embryo as well as lung pathology that can be a sign of aspiration dysphagia in *LgDel* pups.

**Methods:**

Three defined amounts of vitamin A (4, 10, and 16 IU/g) were provided in the maternal diet. Cranial nerve, hindbrain and pharyngeal arch artery development was evaluated in embryos and inflammation in the lungs of pups to determine the impact of altering maternal diet on these phenotypes.

**Results:**

Reduced maternal vitamin A intake improved whereas increased intake exacerbated lung inflammation in *LgDel* pups. These changes were accompanied by increased incidence and/or severity of pharyngeal arch artery and cranial nerve V (CN V) abnormalities in *LgDel* embryos as well as altered expression of *Cyp26b1* in the hindbrain.

**Conclusions:**

Our studies demonstrate that variations in maternal vitamin A intake can influence the incidence and severity of phenotypes in a mouse model 22q11.2 deletion syndrome.

## 1 INTRODUCTION

22q11.2 deletion syndrome (22q11DS) is the most frequent deletion syndrome in humans affecting an estimated one in 4,000 live births (Papangeli & Scambler, 2013). The majority of patients have a three million base pair (3 MB) deletion at chromosome 22q11.2 that includes ~55 protein coding genes (Lindsay, Goldberg & Jurecic et al., 1995; Lindsay, Greenberg & Shaffer et al., 1995; Morrow et al., 1995; Motahari, Moody, Maynard, & LaMantia, 2019). Features of 22q11DS vary widely even in individuals with the same deletion (McDonald‐McGinn et al., 2015). While cardiac and craniofacial dysmorphology as well as a broad range of behavioral disabilities are among the most well‐known features of 22q11DS, almost all 22q11DS patients exhibit some degree of feeding and swallowing problems during childhood, a disorder known as pediatric dysphagia (Dyce et al., 2002; Eicher et al., 2000; Grasso et al., 2018; Jones, Tracy, Perryman, & Arganbright, 2018; Maggadottir & Sullivan, 2013; McDonald‐McGinn et al., 2015; Wong et al., 2019). Infants with 22q11DS gag, regurgitate and aspirate during feedings and have difficulties advancing the volume of feeds (Dyce et al., 2002; Eicher et al., 2000). Aspiration during swallowing is a major factor contributing to frequent respiratory, sinus and ear infections observed in 22q11DS patients (Eicher et al., 2000; Grasso et al., 2018; Jones et al., 2018; Maggadottir & Sullivan, 2013; Wong et al., 2019). Approximately half of children with 22q11DS exhibit difficulties with feeding after the first year of age and a third struggle with advancing to textured foods (Eicher et al., 2000). Approximately one‐third of 22q11DS patients are unable to obtain sufficient nutrients by mouth and require enteral feeding to maintain adequate caloric intake (Eicher et al., 2000). It remains unknown why some patients are able to advance to successfully feed and other requires long‐term supplemental nutrition. Interestingly, feeding difficulties are not correlated with cardiac and craniofacial dysmorphology, suggesting dysphagia is not always a consequence of these malformations (Eicher et al., 2000).

Feeding and swallowing requires coordination of the oral cavity, pharynx and esophagus to move a liquid or solid bolus toward the stomach (Maynard, Zohn, Moody, & LaMantia, 2020). Coordination of swallowing requires successive recruitment and then subsequent inhibition of sensory and motor branches of cranial nerves that connect more than 25 oral, facial, and pharyngeal muscles with swallowing centers in the hindbrain (Matsuo & Palmer, 2008). Disruption of coordinated activity results in aspiration of food and liquids into the nasal cavity and airway, gagging, regurgitation, food refusal, low volume of feeds, and eventually to serious respiratory infections. Pediatric dysphagia can result from anatomic anomalies of the oropharyngeal structures as well as neurological dysfunction that result in weak or uncoordinated movements during swallowing (Matsuo & Palmer, 2008; Rudolph & Link, 2002).

Our previous studies established the *LgDel* mouse model of 22q11DS as an animal model of pediatric dysphagia (Karpinski et al., 2014; LaMantia et al., 2016). *LgDel* pups show diminished growth curves compared to wild type (WT) littermates, aspirate milk into the nasal passages and airway resulting in increased inflammation in the lungs, naso‐sinus and middle ears (Karpinski et al., 2014; LaMantia et al., 2016). Dysphagia in *LgDel* neonates is prefigured by irregularities in the appearance of cranial nerves at embryonic Day 10.5 (E10.5), altered hindbrain gene expression, as well as malformations of structures required for normal feeding such as the palate (Karpinski et al., 2014; LaMantia et al., 2016). Importantly, feeding problems persist into adulthood in the *LgDel* model (Welby et al., 2020).

The cranial nerves and muscles involved in swallowing develop from the pharyngeal arch region of the embryo and require proper patterning of both the hindbrain and pharyngeal arches for coordinated development. Motor neurons whose axons contribute to the cranial nerves are generated in specific hindbrain rhombomeres, as are the neural crest cells that along with cranial placode progenitors, constitute the cranial sensory ganglia (Barlow, 2002; Karpinski et al., 2016). Neural crest cells are specified in precise anterior–posterior domains of the segmented hindbrain and migrate to the first, second and third pharyngeal arches in distinct streams originating from rhombomeres 2, 4, and 6, respectively (Lumsden, Sprawson, & Graham, 1991; Schilling, 2008). In the periphery, neural crest cells interact with a variety of tissues including the paraxial mesoderm that develop into the muscles of mastication and swallowing (Noden & Francis‐West, 2006). Neural crest cells obtain patterning information through reciprocal interactions with tissues encountered before, during and after migration from the rhombomeres (Kulesa, Bailey, Kasemeier‐Kulesa, & McLennan, 2010). Initial cell fate specification in the rhombomeres is dependent on proper establishment of hindbrain patterning influenced by axial patterning signals that include reciprocal gradients of retinoic acid (RA) and fibroblast growth factor 8 (Fgf8) that induce expression of a series of transcription factors involved in specifying rhombomere fate (Diez del Corral & Storey, 2004; Nolte, De Kumar, & Krumlauf, 2019). RA is key for this process, as either too much or too little RA signaling disrupts hindbrain and cranial nerve patterning as well as development of the pharyngeal‐derived structures and muscles involved in feeding and swallowing (Frisdal & Trainor, 2014; Karpinski et al., 2016).

RA is the biologically active metabolite of vitamin A and is obtained from the diet as either provitamin A (e.g., beta‐carotene) or preformed vitamin A. Provitamin A is found in plant‐based foods whereas preformed vitamin A is from animal products and supplements. Provitamin forms of vitamin A require a conversion step that is tightly regulated by the vitamin A status of the individual and excess intake of provitamin A is not typically associated with vitamin A toxicity (Penniston & Tanumihardjo, 2006). Instead, vitamin A toxicity is often due to excessive intake of preformed vitamin A from supplements or foods with high levels of preformed vitamin A such as liver (Penniston & Tanumihardjo, 2006). Because excessive vitamin A during pregnancy is associated with teratogenicity in humans, limits were set recommending that pregnant women restrict intake of preformed vitamin A during pregnancy to no more than 10,000 IU/day if vitamin A deficiency (VAD) is present in the population (WHO, 2011). However, ingestion of certain foods such as liver can provide many times the recommended daily limit, which the developing embryo must buffer in order to allow for normal patterning and development of multiple organ systems.

Either VAD or teratogenic vitamin A exposure results in a spectrum of developmental malformations that overlap with features of 22q11DS (Bailliard & Anderson, 2009; D’Aniello & Waxman, 2015; Hoover, Burton, Brooks, & Kubalak, 2008; LaMantia, 1999; Pan & Baker, 2007; Roberts, Ivins, Cook, Baldini, & Scambler, 2006; Rosa, Wilk, & Kelsey, 1986; Stefanovic & Zaffran, 2017; Tian & Morrisey, 2012). Interestingly, alterations in RA signaling can modify phenotypes in mouse models of 22q11DS (Guris, Duester, Papaioannou, & Imamoto, 2006; Karpinski et al., 2014; Karpinski et al., 2016; Maynard et al., 2013; Ryckebusch et al., 2010; Yutzey, 2010). For example, reduced gene dosage of *Raldh2*, the primary RA synthetic enzyme during establishment of initial hindbrain patterning, can ameliorate some phenotypes in mouse models of 22q11DS including CN V, thymus and fourth arch abnormalities (Guris et al., 2006; Karpinski et al., 2014; Ryckebusch et al., 2010). Here we sought to determine if modest changes in maternal dietary vitamin A intake can similarly alter phenotypes in the *LgDel* mouse model of 22q11DS. Our results indicate changes in maternal vitamin A intake modifies the incidence and severity of abnormalities in cranial nerve branching and PAA formation, alters gene expression in the hindbrain and increases lung inflammation in the *LgDel* model of 22q11DS.

## 2 MATERIALS AND METHODS

### 2.1 Mouse lines and diet supplementation

The *LgDel* mouse line (Meechan et al., 2015; Merscher et al., 2001) is maintained on a congenic C57BL/6NCrl background by mating *LgDel/+* males with females purchased directly from Charles River Laboratories (CN patterning experiment and determination of total liver retinol levels) or first‐generation females generated from crossing C57BL/6NCrl mice purchased from Charles River Laboratories (PAA, hindbrain patterning and lung histology experiments). The standard mouse chow used to maintain mice in our animal facility is the irradiated LabDiet #5 V75 purified diet manufactured by Lab Supply that contains copious amounts of vitamin A (greater than 15 International Units [IU]/gram diet as vitamin A acetate and beta‐carotene; Table 1). All procedures were reviewed and approved by the George Washington University or Children’s National Institutional Animal Care and Use Committees. The purified diets used in this study are based on the AIN‐93G rodent diet and were manufactured by Envigo Teklad Diets (Madison WI; Table 1). The 4 IU diet (TD.06706) contains 4 IU vitamin A per gram of chow as vitamin A Palmitate and is equivalent to the AIN‐93G rodent diet but made with vitamin free casein. vitamin A, D_3_, and E were added back to match levels in the AIN‐93G diet. The purified diets tested include a reduced vitamin A diet (TD.150214) with 2 IU vitamin A per gram of chow as vitamin A Palmitate and vitamin A supplemented diets with 10 IU (TD.150215), 16 IU (TD.150216) and 32 IU (TD.150217) per gram of diet. A different color dye was added to each diet for ease of identification.

**TABLE 1:** Comparison of vitamin content in semipurified versus purified diets used in this study

	Semipurified diet (per kg)	Purified diet (per kg)
Vitamin K (mg)	4.0	0.075
Vitamin B1 (mg)	12	5
Vitamin B2 (mg)	7	6
Vitamin B3 (mg)	94	30
Vitamin B5 (mg)	18	15
Vitamin B6 (mg)	10	6
Vitamin B9 (mg)	2.5	2
Vitamin B12 (mg)	0.051	0.025
Biotin (mg)	0.3	0.2
Vitamin A (IU)	15,000 + beta‐carotene	4,000; 10,000 or 16,000
Vitamin D (IU)	2,200	1,000
Vitamin E (IU)	70	75

For experiments assessing phenotypes, WT C57Bl/6NCrl females were randomly assigned and fed the indicated diets for 2–3 months beginning at weaning and then mated with *LgDel* studs. Females remained on diets until sacrificed when pregnancy reached the indicated embryonic stages or when pups reached postnatal Day 7 (P7). Copulation was verified by the presence of a vaginal plug and designated 0.5 days post coitum (dpc). Yolk sacs or tail clippings were used to genotype embryos as described (Merscher et al., 2001).

To evaluate the impact of diet on total liver retinol levels, livers were removed upon sacrifice from females randomly assigned and fed diets for 3 months beginning at weaning and immediately flash frozen in a dry ice/ethanol bath. Retinoids were extracted with organic solvents and saponified to cleave retinyl esters and other retinoids yielding total free retinol, which was measured by reverse phase HPLC (Eurofins Craft Technologies; Wilson, North Carolina) as described (Craft & Furr, 2019). Statistical significance was calculated by ANOVA with post hoc analysis by Tukey’s test comparing all means to the to the chow diet (online calculator: https://astatsa.com/OneWay_Anova_with_TukeyHSD/). Tukey’s range test was used rather than the Bonferroni correction because of the larger number of multiple comparisons tested in this experiment. The Shapiro–Wilk test was used to confirm the normality of the data (online calculator: http://www.statskingdom.com/320ShapiroWilk.html).

### 2.2 Analysis of pharyngeal arch arteries

India ink injections were done as described (Jianbin et al., 2008; Sugrue, Sarkar, Leatherbury, & Zohn, 2019) using a 1:1 mixture of gelatin (Sigma) and India ink (Pelikan). To visualize pharyngeal arch arteries (PAAs) at midgestation, ink was injected into the left ventricle of embryonic Day 10.5 (E10.5) embryos (32–36 somite stage) using a mouth pipet attached to a pulled glass needle. Representative images were acquired using a Zeiss Lumar microscope with an Axiocam HRc camera (Zeiss) and Axiovision (4.6) software and processed using Adobe Photoshop (14.2). PAA phenotypes were scored blind to genotype by two experienced observers. The frequency of each phenotype was calculated and statistical significance between treatments and groups was determined by chi square analysis (online calculator; http://www.physics.csbsju.edu/stats/) for categorical data.

### 2.3 Quantitation of lung inflammation

The lungs of P7 *LgDel* and WT littermates were collected and fixed in 4% paraformaldehyde overnight at 4°C. Lungs were washed in phosphate‐buffered saline, equilibrated in 30% sucrose and then embedded in optimal cutting temperature (OCT) compound. Serial sections of 20 μm were prepared and stained with Hematoxylin and Eosin (H&E). Images of lungs were obtained using cellSens Dimension software and an Olympus BX63 microscope**.** Quantitation of inflammation was done using Adobe Photoshop and original images corrected for contrast and brightness using methods based on previous studies (Tang, Berman, Swanson, & Yenari, 2010). Briefly, to obtain the inflammation ratio for each image, the number of pixels representing inflamed blood vessels contained in each image was quantitated by setting a threshold value of 145 pixels and dividing by the total area quantified by setting a threshold value of 240 pixels (Supplemental Figure S1). Statistical significance was calculated by ANOVA with post hoc a Bonferroni correction (online calculator: https://astatsa.com). Normality of the data was tested using by the Shapiro–Wilk test (online calculator: http://www.statskingdom.com/320ShapiroWilk.html).

### 2.4 Assessment of cranial nerves

Cranial nerves were visualized in whole E10.5 embryos immunostained for the neurofilament protein (Karpinski et al., 2014) using the mouse anti‐rat neurofilament antibody 2H3 (1:500 dilution) deposited to the DSHB by Tom Jessell and Jane Dodd (Dodd, Morton, Karagogeos, Yamamoto, & Jessell, 1988) and HRP conjugated goat antimouse IgG mouse (Biorad; 1:250 dilution). For analysis of CN V abnormalities, images of each of the three branches (ophthalmic, maxillary and mandibular) were arranged into separate arrays from most to least phenotypically severe by one of the investigators (G. Y.). These arrays were assembled, neither blind to genotype or treatment, based on the length and integrity (normal vs. thin branches, tight bundles versus defasciculated or “fraying” axon bundles) as well as distinct separation from neighboring fascicles for each CN V branch. CN IX/X images were arrayed so that fusion and anastomoses between the two ganglia or nerves could be assessed. CN V anomalies and IX/X fusions were assessed blind to genotype and diet by five independent observers. For the CN V arrays, cutoff points were selected for each image array when branches appeared to deviate from a “typical” to an abnormal phenotype, as well as for abnormal to extreme phenotypes when the anomalies rose to a level where the typical pattern was no longer easily discerned or the relevant branch was nearly or completely absent. CN IX/X fusions required the minimal presence of substantial axon fascicles between the two ganglia or nerves. Statistical significance between treatments and groups was determined by chi square analysis (online calculator: http://www.physics.csbsju.edu/stats/).

### 2.5 Analysis of gene expression in the hindbrain

E9.5 embryos were subjected to in situ hybridization analysis as described (Zohn et al., 2006) with digoxigenin‐labeled antisense probes targeting *Krox20* (Wilkinson, Bhatt, Chavrier, Bravo, & Charnay, 1989) or *Cyp26b1* (Karpinski et al., 2014). Images were acquired using a Zeiss Lumar microscope with an Axiocam HRc camera (Zeiss) and Axiovision (4.6) software and processed using Adobe Photoshop (14.2).

## 3 RESULTS

### 3.1 Altered maternal vitamin A intake and maternal vitamin A status

**FIGURE 1:**
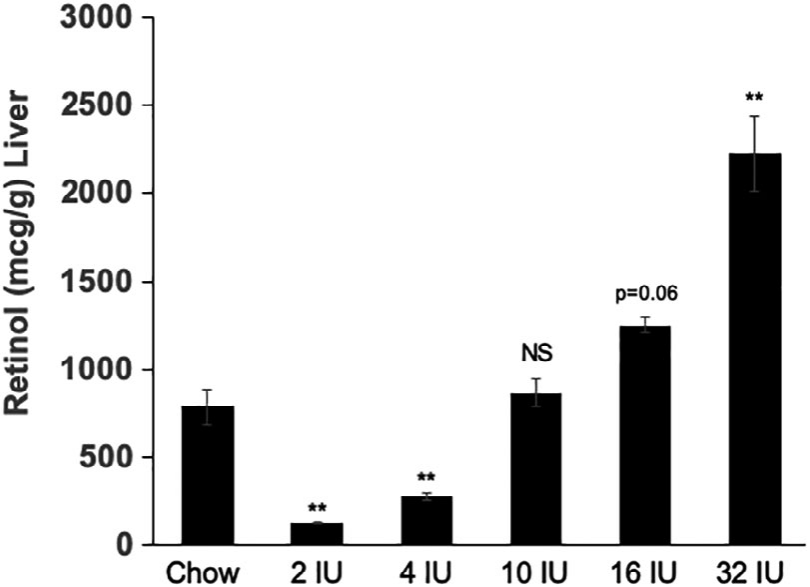
Dose‐dependent changes in maternal liver retinol levels with altered vitamin A content of the maternal diet. Levels of total retinol (mcg/g) were measured in livers from wild type (WT) C57Bl/6 females fed chow or purified diets with defined levels of vitamin A (international units; IU per gram) as retinyl palmitate for 3 months beginning at weaning. There was a dose‐dependent increase in vitamin A status of dams with increasing vitamin A content of the diet. The 10 IU diet resulted in similar liver retinol levels as the chow diet which contains greater than 15 IU vitamin A per gram chow primarily as vitamin A acetate and beta‐carotene**.** The AIN‐93G control diet contains 4 IU vitamin A per gram diet and resulted in a significant reduction in liver retinol levels as compared to dams fed chow or 10 IU diets. Statistical significance was calculated by ANOVA with post hoc analysis by Tukey’s test comparing all means to the to the chow diet. *p <.05; ** *p* <.01

Our previous studies characterizing developmental defects in the *LgDel* mouse line were done in mice fed a chow diet containing greater than 15 IU vitamin A per gram diet as vitamin A acetate and beta‐carotene (Table 1) (Karpinski et al., 2014; Karpinski et al., 2016; Maynard et al., 2013). However, rodent chow is made from natural sources that vary in precise compositions as well as differences in processing the diets and the form of vitamin A provided. Thus, the vitamin A content of chow diets cannot be compared to the content of purified diets. Previous studies demonstrate that levels of liver retinyl esters correlate directly with dietary vitamin A (Batten et al., 2004; Liu, Tang, & Gudas, 2008). Since we hypothesized that maternal vitamin levels were important for modifying phenotypes in the *LgDel* model, we sought to identify a purified diet that results in similar vitamin A liver stores with our feeding protocol. To vary embryonic RA (RA) exposure in utero, wild type C57BL/6NCrl females (hereafter referred to as WT females) were fed diets for 3 months beginning at weaning. Maternal vitamin A status was assessed by measuring total liver retinol levels that include retinyl esters in WT females (*n* = 3; Figure 1). Retinol in livers from WT females fed a chow diet averaged 784 ± 169 μg retinol per gram of liver. A purified diet based on AIN‐93G with 2 IU vitamin A per gram diet as retinol palmitate reduces total liver retinol content by greater than sixfold compared to the chow diet (125 ± 6 μg/g liver; *p* <.01). Feeding the 4 IU diet also significantly reduced liver retinol levels as compared to the chow diet (273 ± 49 μg/g; *p* <.01). In contrast, there was no significant difference between total liver retinol content from females fed the 10 IU vitamin A per gram diet (865 ± 132 μg/g) versus the chow diet. Additional increments of 16 or 32 IU vitamin A per gram diet resulted in dose‐dependent increases in total liver retinol content compared to the chow diet (16 IU: 1,251 ± 70 μg/g, *p* =.06; 25 IU: 2,228 ± 369 μg/g, *p* <.01). Since our goal was to identify a purified diet that, when fed to female mice, results in similar total liver retinol levels as the chow diet used in our previous studies, we designated the 10 IU diet as the “control” diet and utilized the 4 IU and the 16 IU diets to achieve a modest decrease (2.5X) and increase (1.6X) in dietary vitamin A, respectively.

### 3.2 Pharyngeal arch defects with altered maternal dietary vitamin A intake

**FIGURE 2:**
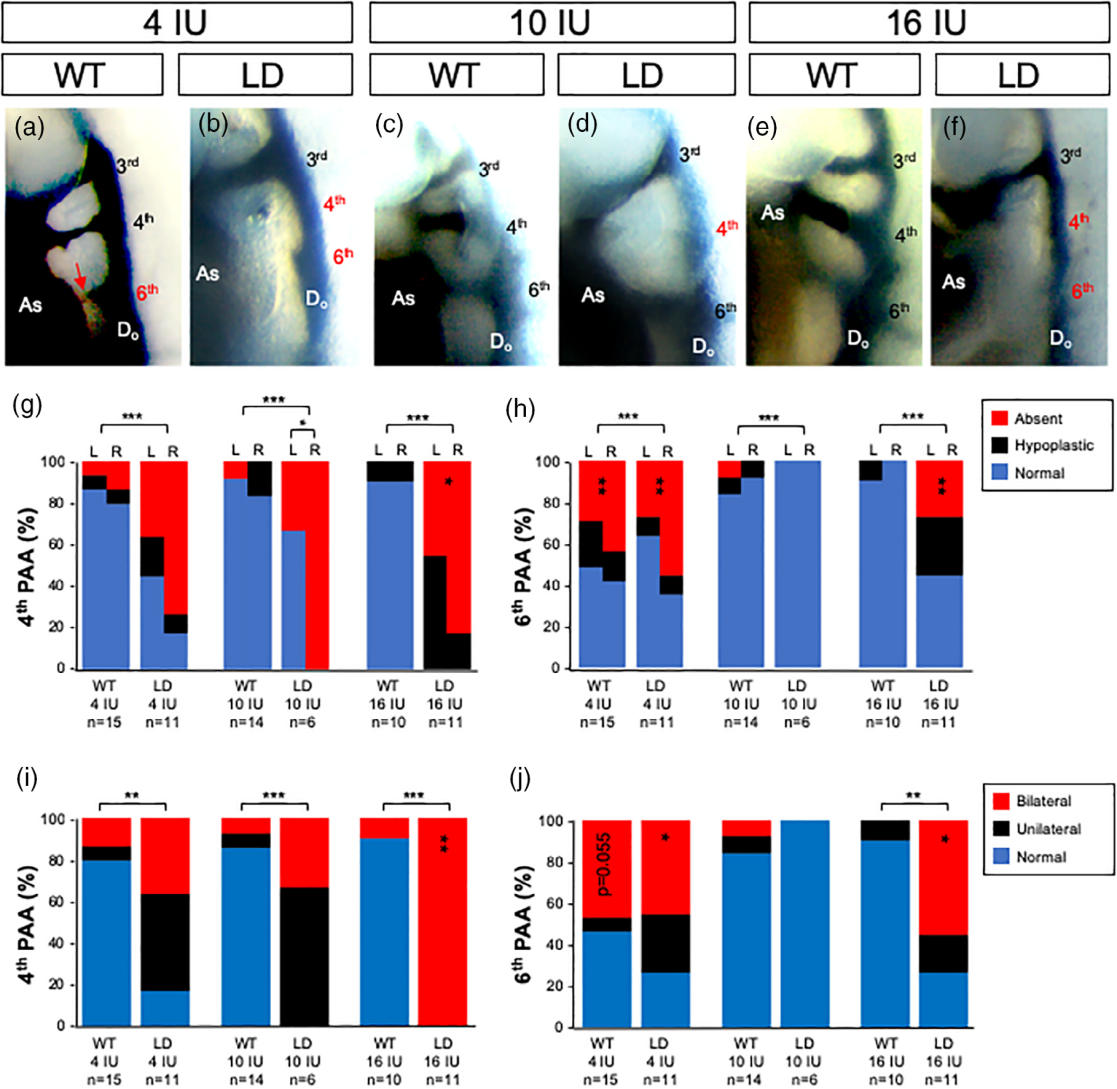
Pharyngeal Arch Artery (PAA) development is altered with changes in vitamin A content of the maternal diet. (a)–(f) Representative right lateral images of E10.5 WT (WT; a, c, e) and *LgDel* (LD; b, d, f) embryos with PAAs visualized by intercardiac India ink injection. Embryos were from dams fed a purified diet containing 4 IU (a, b), 10 IU (c, d) or 16 IU (e, f) vitamin A as retinol palmitate per gram. The number of embryos (*n*) analyzed per treatment is indicated. Hypoplasia (red arrow in panel a) or absence of the fourth and sixth PAAs was the feature scored for quantitative phenotypic analysis and indicated by labels in red. The percentage of normal, hypoplastic or absent fourth (g) or sixth (h) PAAs scored on the left (L) or right (R) is shown for each experimental condition. The percentage of unilateral, bilateral or normal defects for the fourth (i) or sixth (j) PAAs is shown. Statistical significance was determined by chi square and shown as *p <.05; **p <.01 or ***p <.0001. Significant differences between genotypes are indicated by brackets over the bars and within genotypes by vertically aligned asterisks within bars. AS, aortic sac; DoA, dorsal aorta

The fourth pharyngeal arch artery (PAA) in *LgDel* embryos can be modified by altering RA exposure by injection of a subteratogenic dose of all‐transretinoic acid (ATRA) or reducing the gene dosage of *Raldh2*, the primary RA synthetic enzyme expressed in the pharyngeal arch region during PAA development (Maynard et al., 2013; Niederreither et al., 2003). To determine if altering the vitamin A content of the maternal diet has similar effects, we evaluated the impact on formation of the PAAs in *LgDel* embryos and WT littermates from WT dams fed 4, 10, and 16 IU vitamin A per gram diets then mated to *LgDel* males. PAAs in E10.5 embryos were visualized by intracardiac injection of Indian ink (Figure 2). The majority of fourth PAA vessels in *LgDel* embryos (8/12, 67%) from dams fed the 10 IU diet were either hypoplastic or absent with a bias for defects on the left side. Few WT littermates (3/24; 13%) from dams fed the 10 IU diet presented with these abnormalities. The frequency of fourth PAA defects was not significantly altered in *LgDel* (15/22, 68%) or WT embryos (5/30, 17%) from dams fed the 4 IU vitamin A diet. However, feeding dams the 16 IU diet resulted in fully penetrant bilateral fourth PAA anomalies in *LgDel* embryos (22/22, 100%), whereas the frequency of defects was unchanged in WT littermates (2/20, 10%).

No sixth PAA defects were found in *LgDel* embryos (0/12; 0%) and few sixth PAAs were hypoplastic or absent in WT littermates (3/24; 12%) from dams fed the 10 IU vitamin A diet. This finding is consistent with previous studies that indicate the sixth PAA is largely unaffected in mouse models of 22q11DS (Lindsay et al., 2001; Maynard et al., 2013; Ryckebusch et al., 2010; Zhang et al., 2005). However, the frequency of sixth PAA defects was increased (11/22; 50%) in *LgDel* embryos from dams fed the 4 IU vitamin A diet as well as the incidence of bilateral defects (5/11; 47%). The 16 IU diet also increased the incidence of sixth PAA defects in *LgDel* embryos (12/22, 55%) as well as the proportion of embryos with bilateral defects (6/11; 55%). The incidence of PAA defects in WT littermates from dams fed the 4 IU diet was increased from 3/24 (12.5%) on the 10 IU diet to 15/28 (54%) on the 4 IU diet and the increase in bilateral abnormalities approached significance (5/11; 45%; *p* =.05). Unlike *LgDel* embryos, the incidence of sixth PAA defects was not altered in wild type littermates from dams fed the 16 IU diet. These results demonstrate that the sixth PAA is altered in embryos from dams fed the 4 IU diet whereas only *LgDel* embryos are affected when dietary vitamin A content is increased with the 16 IU diet. These findings are reminiscent of our previous studies demonstrating that *LgDel* embryos are more sensitive to acute vitamin A exposures (Maynard et al., 2013).

### 3.3 Increased lung inflammation with altered maternal dietary vitamin A intake

**FIGURE 3:**
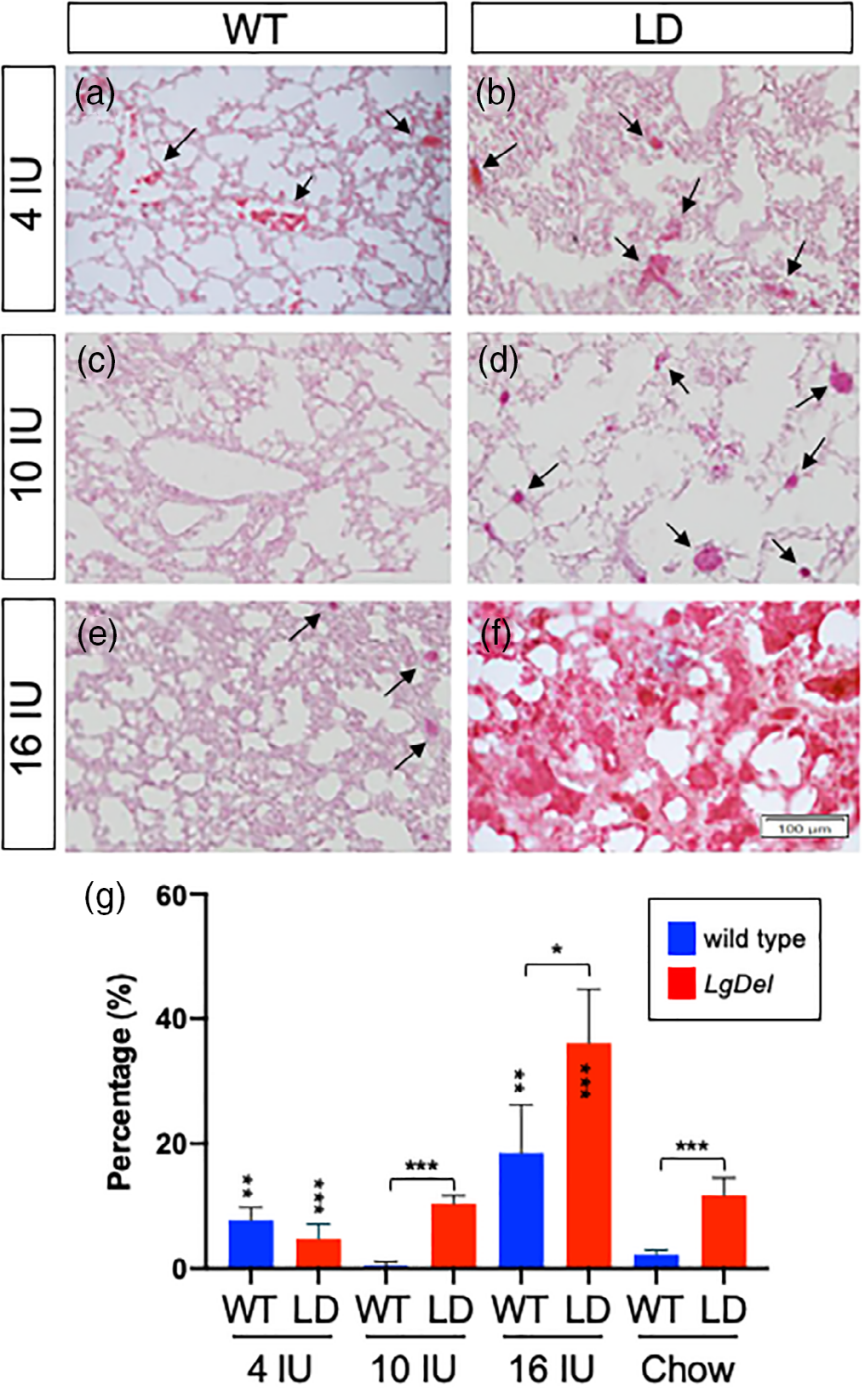
Altering maternal dietary vitamin A intake influences the severity of lung inflammation in the lungs of P7 WT and *LgDel* pups. (a)–(f). Representative images of H&E stained lungs sections from P7 WT (a, c, e) and *LgDel* (LD; b, d, f) pups from dams fed a purified diet containing 4 IU (a, b), 10 IU (c, d) or 16 IU (e, f) vitamin A as retinol palmitate per gram. Dark red staining indicates dilation of blood vessels that occurs with lung inflammation (arrows). Note the dramatic increase in inflamed tissue in the lungs of *LgDel* pups from dams fed the 16 IU diet in panel f. (g) An inflammation ratio was quantified by measuring the area of dark red staining over the total area (see Figure S1 for detailed explanation of method) from five sections averaged from five individuals per group. Statistical significance was determined by ANOVA with a Bonferroni correction for multiple comparisons. Significant differences between genotypes are indicated by brackets over the bars and within genotypes by vertically aligned asterisks within or above bars. *p <.05; **p <.01; ***p <.0001

Our previous studies established aspiration‐based dysphagia resulted in increased lung inflammation, hemorrhage and the presence of milk proteins in the lungs of neonatal *LgDel* pups (Karpinski et al., 2014; LaMantia et al., 2016). While increased lung inflammation and hemorrhage can result from altered immune system function in 22q11DS (Sullivan, 2019) and VAD can lead to histopathological changes in the lungs (Timoneda et al., 2018), the association with milk proteins and other signs of dysphagia in this mouse model suggest aspiration is a contributing factor (Karpinski et al., 2014; LaMantia et al., 2016). To evaluate if varying maternal dietary vitamin A during pregnancy alters the incidence or severity of lung inflammation and hemorrhage in pups, WT dams were fed 4, 10, or 16 IU vitamin A diets then mated to *LgDel* males. Lungs of the offspring were assessed histologically for signs of inflammation at postnatal Day 7 (P7; Figure 3). As in our previous studies in mouse pups from dams fed a chow diet (Karpinski et al., 2014; LaMantia et al., 2016), the lungs of *LgDel* pups from dams fed the 10 IU diet show significantly greater signs of inflammation than WT littermates. 10% of the lung area showed hemorrhages in *LgDel* pups from dams fed the 10 IU diet, whereas negligible areas of inflammation (0.41%) were detected in WT littermates. Hemorrhages were significantly reduced in *LgDel* pups from dams fed the 4 IU vitamin A diet (4.7%) compared to the 10 IU diet; however, there was no statistically significant difference between *LgDel* and WT littermates from dams fed the 4 IU diet, likely due to increased lung inflammation observed in WT pups (4 IU: 7.74% vs. 10 IU: 0.41%). Similarly, lung inflammation was increased in *LgDel* pups (36.1%) as well as in WT littermates (18.5%) from dams fed the 16 IU vitamin A diet; however, the increase in inflammation was greater in *LgDel* pups. Together, these data indicate that lung inflammation, a possible indicator of aspiration‐based dysphagia in *LgDel* pups (Karpinski et al., 2014; LaMantia et al., 2016), is sensitive to changes in maternal vitamin A intake in both *LgDel* pups and WT littermates.

### 3.4 Maternal dietary vitamin A intake and cranial nerve development

**FIGURE 4:**
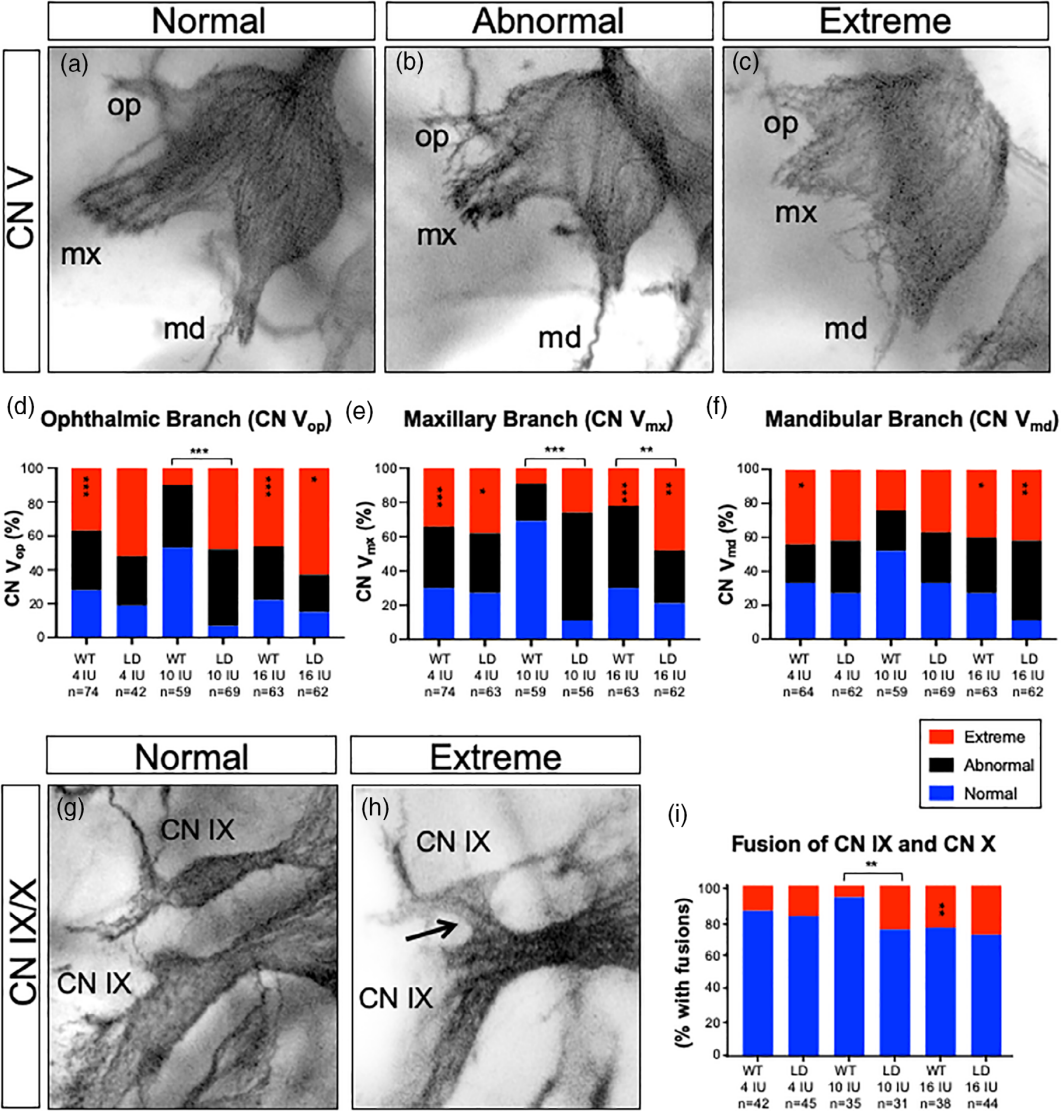
Vitamin A content of the maternal diet alters the incidence and severity of cranial nerve abnormalities in both *LgDel* embryos and WT littermates. (a)–(c) Representative images showing typical examples of CN V scored as (a) normal, (b) abnormal or (c) extreme. (d)–(f). Quantitation of abnormalities of the ophthalmic (CN V_op_, panel d) maxillary (CN V_mx_, panel e) and mandibular (CN V_md_, panel f) branches of CN V were scored separately in E10.5 *LgDel* (LD) embryos and WT littermates from dams fed a purified diet containing 4 IU, 10 IU or 16 IU vitamin A as retinol palmitate per gram. (g, h) Representative images of CN IX/X scored as (g) normal or (h) extreme when fused. (i) Quantitation of CN IX/X fusions. Statistical significance was determined by chi square. *p <.05; **p <.01; ***p <.0001

Our previous studies demonstrate that disrupted development of CN V and CN IX/X prefigures feeding and swallowing difficulties in the *LgDel* model (Karpinski et al., 2014; Karpinski et al., 2016; LaMantia et al., 2016). Improved lung inflammation in *LgDel* pups from dams fed the 4 IU diet was interesting in light of our previous findings that CN V anomalies, but not fusion of CN IX/X were rescued by reducing *Raldh2* gene dosage, the primary RA synthetic enzyme, which reduces RA signaling in the embryo by approximately 35% (Karpinski et al., 2014; Karpinski et al., 2016; LaMantia et al., 2016; Maynard et al., 2013). To evaluate the impact of altered maternal vitamin A intake on CN V development in offspring, we assessed morphological differentiation of the three main branches of CN V: ophthalmic (CN V_op_), maxillary (CN V_mx_) and mandibular (CN V_md_). Each branch was given a designation of normal if it matched an “index” WT control, “abnormal” if it deviated moderately from that control or “extreme” if the dysmorphology resulted in a CN V branch that was either largely absent or unrecognizable (Figure 4).

The ophthalmic branch of CN V (CN V_op_) provides sensory innervation to the skin around the eye, the cornea and structures derived from the frontonasal prominence (Huff & Daly, 2019). CN V_op_ branches that appear frayed and defasciculated were scored as abnormal and truncated and absent branches were scored as extreme. The incidence and severity of CN V_op_ abnormalities was greater in *LgDel* versus WT littermates from dams fed the 10 IU diet (*p* <.001; Figure 4D). Indeed, few of the CN V_op_ scored in *LgDel* embryos from dams fed any of the diets were scored “normal” (*n* = 5/64; 7.8%). The proportion of *LgDel* embryos from dams fed the 16 IU diet with extreme CN V_op_ phenotypes was significantly increased over *LgDel* embryos from dams fed the 10 IU diet (63% *LgDel* 16 IU vs. 48% *LgDel* 10 IU; *p* <.05). In contrast, CN V_op_ abnormalities were significantly increased in WT littermates from dams fed either 4 IU (36%) or 16 IU (46%) diets (*p* <.001) compared to the 10 IU diet.

The maxillary branch of CN V (CN V_mx_) innervates the upper jaw, lip, and teeth, the naso‐pharyngeal mucosa, muscles of the upper palate and the skin of the midface (Shafique, 2019). Defects were classified as abnormal if CN V_mx_ appeared as disorganized, frayed bundles or extreme if branches were severely truncated or missing. As shown in Figure 4E, the overall incidence of CN V_mx_ branches scored as abnormal was decreased from 89% to 73% in *LgDel* embryos fed the 4 IU diet; however, the proportion of those anomalies classified as extreme increased from 27% to 38% (*p* <.05). A similar pattern was found in embryos from dams fed the 16 IU diet when compared to embryos from dams fed the 10 IU diet, where the overall incidence of defects decreased from 89% to 79% but the incidence of extreme defects increased from 27% to 48% (*p* <.01). The incidence and severity of affected CN V_mx_ branches also increased in WT littermates from dams fed either the 16 IU or 4 IU compared to the 10 IU diet (*p* <.001). The increase in CN V_mx_ defects in WT littermates from dams fed the 4 IU diet was so great that the difference in phenotypic frequencies between *LgDel* and WT littermate become negligible. In contrast, the incidence of defects between *LgDel* embryos and WT littermates from dams fed the 16 IU diet was significantly increased (*p* <.01). Thus, similar to CN V_op_, CN V_mx_ development in *LgDel* embryos is sensitive to changes in maternal vitamin A intake in both *LgDel* embryos and WT littermates.

The mandibular branch of CN V (CN V_md_) innervates the muscles of mastication and the tongue (Ghatak & Ginglen, 2019). The frequency of CN V_md_ anomalies was not significantly changed between *LgDel* and WT littermates from dams fed the 10 IU diet (*p* <.01; Figure 4F). However, the incidence of CN V_md_ abnormalities increased from 67% to 89% (*p* <.01) in *LgDel* embryos from dams fed the 16 IU diet. Similarly, defects of CN V_md_ were increased in WT embryos from dams fed either the 4 IU or 16 IU diets from 48% to 67% or 73% (*p* <.05), respectively. Thus, defects in development of CN V_md_ were significantly impacted in *LgDel* embryos from dams fed increased but not decreased vitamin A, whereas WT littermates were sensitive to both increased and decreased vitamin A exposures.

Finally, we assessed the impact of maternal diet on fusion of CN IX and CN X that influence pharyngeal and laryngeal function during swallowing as well as palate and tongue sensory‐motor control (Gillig & Sanders, 2010). The incidence of fusions of CN IX and CN X was greater in *LgDel* versus WT littermates from dams fed the 10 IU diet (*p* <.01; Figure 4G, I). The frequency of CN IX/X fusions was not significantly altered in *LgDel* embryos between diets. But, fusions were more common in WT littermates from dams fed the 16 IU vitamin A diet (*p* <.01). Thus, CN IX/X is largely unaffected by altered maternal dietary exposure in *LgDel* embryos; but more frequent in WT littermates exposed to higher vitamin A levels.

### 3.5 Maternal vitamin A intake and hindbrain gene expression

**FIGURE 5:**
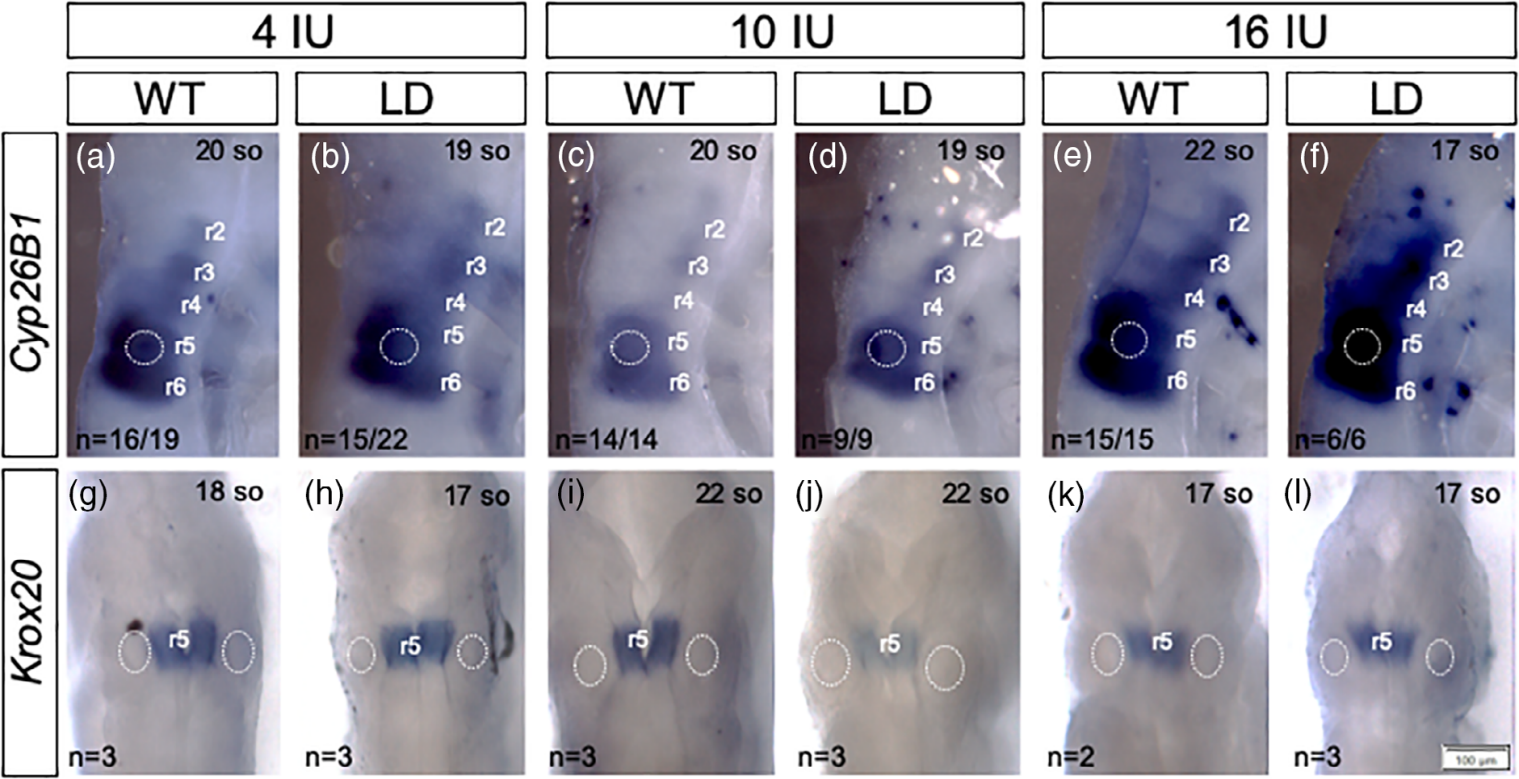
Changes in vitamin A content of the maternal diet alters gene expression in the hindbrain. Expression of *Cyp26b1* (a)–(f) and *Krox20* (g)–(l) were assessed by whole mount in situ hybridization in the hindbrain of E9.5 *LgDel* (LD) embryos and WT (WT) littermates (between 17 and 22 somite stages) from dams fed a purified diet containing 4 IU, 10 IU or 16 IU vitamin A as retinol palmitate per gram. (a)–(f) *Cyp26b1* is expressed in r5 and r6 and ventrally in r2–r4. Representative images showing increased expression in both *LgDel* embryos and WT littermates from dams fed the 4 IU diet. Expression is increased further with the 16 IU diet where expression in r4 expands dorsally in *LgDel* embryos. (g, h) Expression of *Krox20* in r5 was reduced in *LgDel* embryos from dams fed the 10 IU diet and expression was restored with altered maternal vitamin A intake. The number of embryo that presented with the expression pattern shown is indicated as well as the somite stage (so). The otic vesicle is positioned normally adjacent to r5 and r6 in all embryos examined (outlined by the white dotted line)

Cranial nerve anomalies can reflect altered hindbrain patterning, which requires establishment of a precise RA gradient in the hindbrain (Frisdal & Trainor, 2014; Nolte et al., 2019). To evaluate whether altering vitamin A via maternal diet significantly impacts RA signaling in the hindbrain, we evaluated expression of *Cyp26b1*. *Cyp26b1* expression is regulated by RA signaling as part of a feedback loop to normalize fluctuating RA levels and can be a sensitive readout of altered RA signaling in the hindbrain (Lee et al., 2012; Reijntjes, Blentic, Gale, & Maden, 2005; White & Schilling, 2008). Furthermore, our previous studies demonstrate *Cyp26b1* expression is increased in rhombomeres 2 through 4 (r2‐4) of the hindbrain in *LgDel* embryos (Karpinski et al., 2014; LaMantia et al., 2016). As in embryos from dams fed chow diets (MacLean et al., 2001; White & Schilling, 2008), E9.5 embryos from dams fed the 10 IU diet showed *Cyp26b1* expression in r5 and r6, with lower levels of expression in ventral domains of r2‐4. *LgDel* embryos from dams fed the 10 IU diet show elevated *Cyp26b1* expression (Figure 5A‐H) as compared to WT littermates. Expression was expanded and intensified in the majority (70–85%) of *LgDel* as well as WT littermates from dams fed the 4 IU diet. The remaining embryos exhibited expression patterns similar to those from dams fed the 10 IU diet (not shown). *Cyp26b1* expression was also much higher in the hindbrain of *LgDel* and WT littermates from dams fed the 16 IU diet, with even greater increases in the *LgDel* hindbrain expanding dorsally in r4 (Figure 5A‐H). These results indicate that altered maternal vitamin A intake impacts expression of the RA buffering machinery in the developing embryo.

To begin to evaluate if changes in maternal vitamin A intake alters hindbrain rhombomere patterning, expression of the zinc finger transcription factor *Krox20* was visualized by whole mount in situ hybridization. *Krox20* is initially expressed in r3 and r5 in the E8.5 mouse embryo but expression is not maintained in r3 and by E9.5 *Krox20* expression becomes restricted to r5 (Sham et al., 1993; Swiatek & Gridley, 1993). *Krox20* expression was reduced in *LgDel* embryos compared to WT littermates from dams fed the 10 IU diet (Figure 5G‐L). Expression of *Krox20* in *LgDel* embryos was restored when dams were fed the 4 and 16 IU diets, but altering maternal diet had no effect on *Krox20* expression in WT littermates (Figure 5G‐L). Thus, alterations in expression of *Cyp26b1* (a marker for altered RA signaling) but not *Krox20* (a marker for altered segmentation of the hindbrain) in response to changes in maternal diet parallels abnormalities in CN organization and lung inflammation observed in both *LgDel* embryos and WT littermates.

## 4 DISCUSSION

Modifiable environmental factors such as maternal diet are of great interest as contributors to birth defects as well as prevention. Either VAD or teratogenic RA exposure results in a spectrum of developmental malformations that overlap with 22q11DS (Bailliard & Anderson, 2009; D’Aniello & Waxman, 2015; Hoover et al., 2008; Pan & Baker, 2007; Rosa et al., 1986; Stefanovic & Zaffran, 2017; Tian & Morrisey, 2012). RA is the biologically active metabolite of vitamin A and plays key roles in multiple stages of development of the structures and neural circuits involved in feeding (Frisdal & Trainor, 2014; Maynard et al., 2020). Our focus on vitamin A as a modifier for pediatric dysphagia in this study was prompted by previous studies demonstrating interactions between altered RA signaling and the incidence and severity of phenotypes in mouse models of 22q11DS. Notably, reduction of RA synthesis by loss of a single copy of *Raldh2* reduces embryonic RA signaling by 35% and can improve CN V development in the *LgDel* model (Karpinski et al., 2014; LaMantia et al., 2016; Maynard et al., 2013). Similarly, reduced *Raldh2* gene dosage improved aortic arch development in other mouse models of 22q11DS (Guris et al., 2006; Ryckebusch et al., 2010). We now demonstrate that reducing the vitamin A content of the maternal diet 2.5 fold by feeding dams a purified diet with 4 IU per gram vitamin A can improve lung inflammation in *LgDel* pups. However, not all phenotypes were improved by feeding the 4 IU diet. For example, while the overall incidence of CN V_mx_ abnormalities was decreased, the severity of defects worsened. Similarly, the incidence of sixth PAA defects were increased in both WT and *LgDel* embryos. Thus, while improving some phenotypes, reducing vitamin A intake may have pleotropic effects differentially impacting distinct features of 22q11DS. Furthermore, our data suggest reducing vitamin A intake has negative impacts on development of WT embryos—at least on the C57BL/6NCrl mouse background used in our study.

On the other hand, we found that elevating maternal vitamin A intake by a modest 1.6‐fold consistently increased the severity of all phenotypes examined in *LgDel* embryos but not wild type littermates. Notably, there was a dramatic increase in lung inflammation in *LgDel* pups from dams fed the higher vitamin A diet. The severity of CN V_mx_ defects increased, as did the incidence and severity of CN V_md_ abnormalities. Expression of *Cyp26b1* was increased in r2‐r6 and expanded dorsally in r4. In agreement with our previous studies showing increased severity of PAA defects with subteratogenic RA exposure (Maynard et al., 2013), bilateral PAA defects became fully penetrant in *LgDel* embryos from dams fed the 16 IU diet during pregnancy. These data demonstrate that a modest increase in maternal vitamin A intake during pregnancy preferentially impacts development of the *LgDel* embryo.

In our experiments we tested the impact of modest changes in vitamin A content using 10 IU as our “control” diet as this diet resulted in similar vitamin A liver stores as the chow diet used in our previous studies (Karpinski et al., 2014; Karpinski et al., 2016; Maynard et al., 2013). A 4 IU vitamin A diet was designated as the “low” and 16 IU as the “high” vitamin A diet in our study. Interestingly, our results indicate that the WT background strain (C57BL/6NCrl) used is surprisingly sensitive to changes in vitamin A exposure. The lungs of WT pups from dams fed either normal chow or the 10 IU diet exhibited remarkably low levels of inflammation which increased with elevations or reductions in maternal vitamin A intake. It is possible that these observed effects on lung inflammation may be due to factors in addition to aspiration‐based dysphagia. For example, VAD results in changes in the pulmonary epithelium that predispose to dysfunction and respiratory disease (Timoneda et al., 2018). However, the histological appearance of these VAD‐induced lung changes consist of delayed development with poorly developed bronchial passages and reduced branching (Antipatis et al., 1998) and are very different than the inflammation quantified in our experiments. Importantly, the low vitamin A diet used in our study is equivalent to the control diet used in this and other studies examining the impact of VAD on lung pathology (Antipatis et al., 1998; Baybutt, Hu, & Molteni, 2000; Schuster, Kenyon, & Stephensen, 2008).

CN V development in WT littermates were also sensitive to changes in the vitamin A content of the maternal diet. We found that both the incidence and severity of CN V defects in WT embryos occurred at frequencies akin to those observed in *LgDel* littermates from dams fed the 10 IU diet. Analogous to *LgDel* littermates, expression of *Cyp26b1* was also increased in WT embryos in response to both increased and decreased maternal intake. While the fourth PAA was unaffected by maternal diet in WT littermates, development of the sixth PAA was sensitive to reduced maternal vitamin A intake. The spectrum of abnormalities observed in WT littermates in the 4 IU vitamin A diet group were quite surprising as this is the control AIN‐93G diet used in a variety of embryological studies investigating the impact of maternal nutrition on embryonic development (see [Beaudin et al., 2012; Beaudin et al., 2011; Beaudin, Perry, Stabler, Allen, & Stover, 2012; Dickman, Thaller, & Smith, 1997; Freire et al., 2011; Kane, Folias, & Napoli, 2008; MacFarlane et al., 2009; Obrochta, Kane, & Napoli, 2014; Reeves, 1997; Reeves, Nielsen, & Fahey Jr., 1993 Reeves, Rossow, & Lindlauf, 1993] for examples). Importantly, our measurements of maternal vitamin A stores in livers confirms that the AIN‐93G diet leads to reduced vitamin A stores in the dams as compared to feeding a chow diet. Thus, this level of dietary vitamin A does not meet the needs for development of WT embryos of the C57BL/6NCrl mouse background used in our study.

Our analysis focused on the initial development of the cranial nerves that control swallowing. However, swallowing is a complex process and structures in addition to the cranial nerves likely also contribute to oropharyngeal dysfunction in 22q11DS. These include deformities of the digestive tract such as tracheoesophageal fistula, esophageal atresia and tracheal atresia. Abnormalities of the aortic arch such as the presence of a vascular ring can impinge upon the trachea or esophagus interfering with swallowing. Craniofacial defects that complicate swallowing are common in 22q11DS and include micrognathia, velopharyngeal insufficiency, cleft lip, and palate. While our data demonstrate CN V and CN IX/X are compromised in *LgDel* embryos (Karpinski et al., 2014; Karpinski et al., 2016; LaMantia et al., 2016) and abnormalities of CN V are modified by maternal vitamin A intake, abnormalities of additional cranial nerves as well as further development of CN V, CN IX, and CN X are also relevant for aspiration‐based dysphagia. Finally, our previous work identified changes in mature hypoglossal motor neuron function associated with dysphagia in the *LgDel* model suggesting that cranial motor nerve function is also impacted in *LgDel* mice (Wang, Bryan, LaMantia, & Mendelowitz, 2017). Whether this is a result of the divergent early patterning (Karpinski et al., 2014; Karpinski et al., 2016; LaMantia et al., 2016) influencing subsequent development under the influence of the RA pathway or additional unrelated consequences of diminished 22q11.2 gene dosage at later stages remains to be determined.

Either VAD or excess vitamin A causes a variety of developmental abnormalities similar to 22q11DS and RA levels and signaling must be tightly regulated during embryogenesis. This is accomplished through engagement of positive and negative feedback loops that alter expression of the enzymes that control availability of the active RA ligand as well as subsequent signaling (D’Aniello & Waxman, 2015; Shannon, Moise, & Trainor, 2017). Teratogenesis occurs when this buffering capacity is overrun (D’Aniello & Waxman, 2015). This is best studied in the Zebrafish embryo where exposure to high doses of RA results in increased expression of RA degradation enzymes (e.g., *Cyp26a1*) and decreased expression of RA synthesis enzymes (e.g., *Raldh2* and *Rdh10*) (D’Aniello, Rydeen, Anderson, Mandal, & Waxman, 2013). Interestingly, when the buffering capacity of this system is overrun, feedback loops become hyperactive, resulting in a “pseudo vitamin A‐deficient” state (D’Aniello & Waxman, 2015; Shannon et al., 2017). Similar paradoxical teratogenic mechanisms are reported in mice where exposure to high levels of RA initially result in increased expression of RA responsive genes, then subsequent feedback loops engage that over‐repress RA signaling leading to phenotypes consistent with VAD (Lee et al., 2012). This model nicely explains the similarities in phenotypes caused by RA teratogenesis and deficiency and could explain our paradoxical finding that *Cyp26b1* expression was elevated by both increased and decreased maternal vitamin A intake.

The recommended daily allowance for vitamin A intake during pregnancy in humans is less than 10,000 IU/day (WHO, 2011). But pregnant women may transiently encounter even higher levels of dietary vitamin A depending upon food preferences and levels of vitamin A intake may vary substantially during pregnancy. Our mouse study indicates even small increases in maternal vitamin A intake may pose severe challenges for the *LgDel* embryo and further human studies would be needed to determine if vitamin A content of the maternal diet might have similar effects in 22q11DS patients.

## Supporting information


**FIGURE S1** Method developed for quantification of inflammation in lung tissue. A, B. Images of H&E stained lung sections were manipulated in Adobe Photoshop to quantitate regions of inflammation. C, D. Using the “Threshold” function, a value of 240 pixels was set to obtain the total area of lung tissue in the image. E, F. To quantitate inflamed blood vessels in the image, a threshold value of 145 pixels was set. The percentage of inflamed tissue was calculated by dividing the inflamed by total pixels and multiplying by 100.

## Data Availability

The data that support the findings of this study are available from the corresponding author upon reasonable request.
