# Supplementary figures and images for: Variations in maternal vitamin A intake modifies phenotypes in a mouse model of 22q11.2 deletion syndrome
Source: Birth Defects Res. 2020 May 20;112(16):1194–208. doi: 10.1002/bdr2.1709 (PMC7586978; doi:10.1002/bdr2.1709)

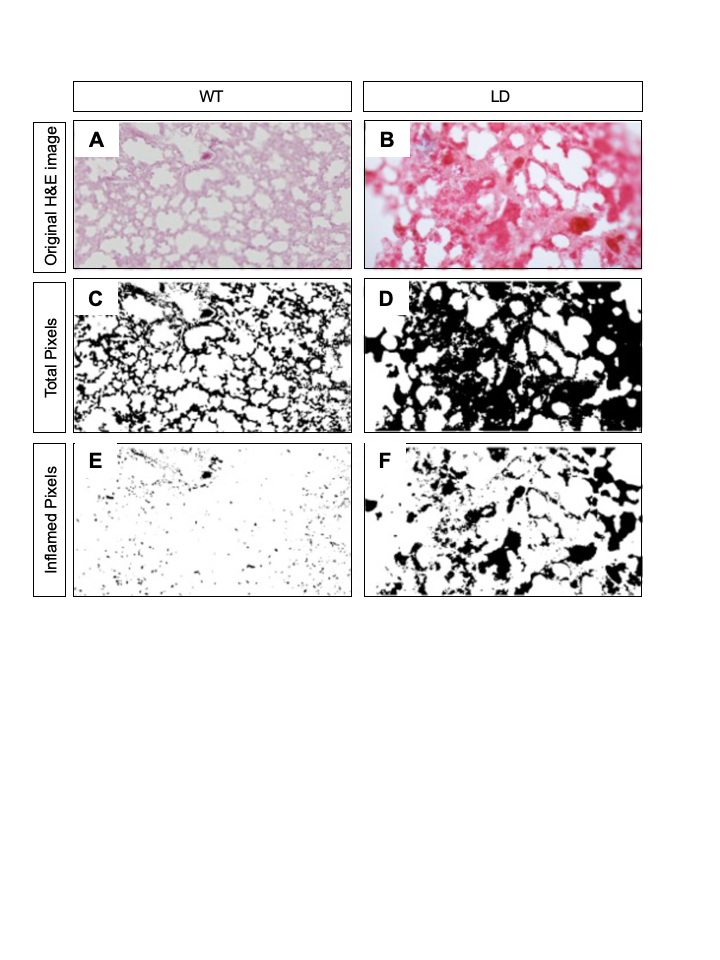

Supplement: Supplementary file 1 — FIGURE S1 Method developed for quantification of inflammation in lung tissue. A, B. Images of H&E stained lung sections were manipulated in Adobe Photoshop to quantitate regions of inflammation. C, D. Using the “Threshold” function, a value of 240 pixels was set to obtain the total area of lung tissue in the image. E, F. To quantitate inflamed blood vessels in the image, a threshold value of 145 pixels was set. The percentage of inflamed tissue was calculated by dividing the inflamed by total pixels and multiplying by 100. [file BDR2-112-1194-s001.tiff]
